# Total knee arthroplasty after anterior cruciate ligament reconstruction with the use of image-based robotic technology and functional alignment

**DOI:** 10.1051/sicotj/2025025

**Published:** 2025-05-19

**Authors:** Christos Koutserimpas, Luca Andriollo, Pietro Gregori, Enejd Veizi, Reha Tandogan, Sébastien Lustig, Konstantinos Dretakis

**Affiliations:** 1 2nd Department of Orthopaedic Surgery, “Hygeia” General Hospital of Athens 151 23 Greece; 2 School of Rehabilitation Health Sciences, University of Patras 265 04 Greece; 3 Orthopaedics Surgery and Sports Medicine Department, FIFA Medical Center of Excellence, Croix Rousse Hospital, Hospices Civils de Lyon, Lyon North University Hospital 103 Grande Rue de la Croix Rousse 69004 Lyon France; 4 Ortopedia e Traumatologia, Fondazione Poliambulanza 25124 Brescia Italy; 5 Fondazione Policlinico Universitario Campus Bio-Medico Via Alvaro del Portillo, 200 00128 Roma Italy; 6 Ankara Bilkent City Hospital, Department of Orthopedics and Traumatology Ankara 06800 Turkey; 7 Ortoklinik & Çankaya Hospital Ankara 06700 Turkey; 8 Univ Lyon, Claude Bernard Lyon 1 University, IFSTTAR, LBMC UMR_T9406 69622 Lyon France

**Keywords:** Robotic knee, Functional knee positioning, Functional alignment, Personalized alignment, Total knee

## Abstract

*Background*: Total knee arthroplasty (TKA) in patients with prior anterior cruciate ligament reconstruction (ACLR) presents unique challenges due to altered knee kinematics, residual instability, and fixation implants that may interfere with implant positioning. Image-based robotic-assisted TKA enables the functional alignment (FA) strategy that accounts for individual bony anatomy, ligamentous laxities, and anterior compartment characteristics. *Surgical technique*: This technique involves a CT-based robotic workflow where femoral and tibial components are planned based on patient-specific alignment and soft tissue balance. Intraoperative assessment with a digital tensioning device guides fine-tuning of flexion and extension gaps, ensuring balanced implant positioning while minimizing soft tissue releases. Fixation implants from prior ACLR are identified using robotic navigation, allowing for targeted adjustments such as selective removal or controlled elevation of components to avoid excessive bone loss. Patellar tracking is dynamically evaluated with a probe, facilitating real-time adjustments to optimize mediolateral tracking and anterior offset. *Discussion*: Given the altered biomechanics in post-ACLR knees, FA may provide a physiological alignment by accommodating native laxities and reducing the risk of residual instability. Additionally, robotic guidance allows for precise management of fixation implants, ensuring optimal implant positioning and bone preservation. While further studies are needed, robotic-assisted FA represents a promising approach for enhancing outcomes in TKA for post-ACLR patients.

## Introduction

Patients with a history of anterior cruciate ligament reconstruction (ACLR) present unique challenges in total knee arthroplasty (TKA) due to altered knee kinematics, potential residual instability, and the presence of fixation implants that may interfere with implant positioning [1, 2]. Addressing these complexities requires a precise and individualized approach. Image-based robotic-assisted TKA had proven to be a safe, reliable, and accurate technique that enables the functional alignment (FA) strategy that accounts for each patient’s bony anatomy, ligamentous laxities, and anterior compartment characteristics to optimize implant placement and knee balance [3–7]. By leveraging robotic technology, surgeons can achieve a three-dimensional alignment that preserves native knee kinematics while accommodating prior ACLR-related changes.

There is limited guidance on managing the unique challenges of TKA in patients with prior ACLR, particularly regarding hardware interference and altered knee kinematics. This technique illustrates how image-based robotic-assisted TKA with FA can address these issues. A cohort of patients is also presented to support the feasibility of this approach in clinical practice.

## Methods

A consecutive cohort of patients with previous ACLR undergoing image-based TKA with the FA principles were evaluated retrospectively from June 2021 to December 2022. Demographics are presented in Table 1, while the Knee Society Score (KSS)-knee and -function were assessed pre- and post-operatively to validate the presented technique.

**Table 1:** The demographics of the studied cohort are presented. Data are reported as mean values with standard deviations (SD).

Cohort = 25 patients	
Age	61.8 (SD = 8.02)
Gender (Male)	*N* = 12 (48%)
BMI	24.99 (SD = 3.4)
Follow-up	35.44 (SD = 6.71)

The study was approved by the Scientific Committee of Hygeia Hospital (Ref. No. 663, 20/12/2023) and conducted in accordance with the Declaration of Helsinki.

### Surgical technique

The patient is placed in a standard supine position, with one arm resting on a lateral support in abduction and the other on the surgical table. The leg is positioned in a leg holder that allows unrestricted movement from full extension to full flexion (Video 1).

### FA workflow

The FA technique aims to achieve a balanced three-dimensional implant positioning by considering the patient’s unique bony anatomy, ligamentous laxities, and anterior compartment characteristics [8, 9].

After joint exposure, checkpoints are placed, and anatomical reference points are registered. Real-time intraoperative assessment of coronal alignment and soft tissue balance is performed at full extension, 90° flexion, and maximum flexion. Ligamentous laxities are evaluated using varus and valgus stress maneuvers in extension and in flexion. Adjustments to femoral and tibial cuts are made to achieve optimal balance. The anterior compartment is assessed to maintain native trochlear morphology and avoid overstuffing.

When utilizing an image-based robotic platform, the FA workflow begins with CT-based preoperative planning, where femoral and tibial implant models are digitally superimposed onto the patient’s CT images.

Coronal alignment is planned to maintain native epiphyseal orientation by equalizing medial and lateral resections and correcting for bone wear as needed. We follow a “restricted” FA strategy with the tibial component’s coronal positioning being set within a safe range of 3° varus to 2° valgus (with reference to the femoral mechanical axis), depending on the patient’s overall limb alignment. Sagittal positioning is determined by matching the individual’s native posterior tibial slope, with adjustments based on the selected implant type. For cruciate-retaining (CR) designs, the posterior slope can be set between 0° and 3°, depending on patient-specific anatomy, while for posterior-stabilized (PS) designs, it is typically maintained between 0° and 1° to optimize kinematics and implant stability. Axially, the tibial component is aligned with the tibial anteroposterior axis, ensuring optimal medio-lateral and AP bone coverage.

For the femoral component, coronal alignment is planned within a range of 3° valgus to 3° varus (with reference to the tibial mechanical axis), preserving the patient-specific epiphyseal orientation. Distal and posterior femoral resections are set to maintain the medial joint line height in both flexion and extension while accounting for implant thickness. In the axial plane, femoral rotation is optimized by referencing the transepicondylar axis (TEA) or the posterior condylar axis (PCA), with strict attention to reproducing the patient’s native trochlear groove as visualized on the preoperative CT scan. While no strict rotational boundaries are enforced, accurate trochlear alignment is crucial to avoid mismatch. Sagittal positioning is refined to prevent anterior notching, which can be mitigated by increasing femoral flexion or anterior translation when necessary. The limit for combined flexion (tibial slope and femoral flexion) is 10°.

Intraoperatively, joint balancing is performed using a digital tensioning device to assess soft tissue laxity in both extension and flexion under controlled stress. Adjustments to femoral and tibial component alignment are made in all three dimensions to achieve optimal balance within predefined parameters. The goal is to achieve flexion and extension gaps within 0–1.5 mm, depending on whether a CR or PS design is used, while maintaining an additional 1–2 mm of lateral compartment laxity in flexion to replicate natural knee mechanics. Final implant selection (CR or PS) is determined based on posterior cruciate ligament integrity and intraoperative flexion stability, with trial components used to confirm alignment before definitive implantation.

### Image-based guidance and the detection of fixation implants

Image-based robotic-assisted TKA provides a precise method for planning and executing bone resections while accommodating pre-existing hardware from ACLR. The preoperative CT scan allows for the identification of fixation implants, such as interference screws or staples, and aids in strategizing component positioning to minimize disruption of existing structures. Intraoperatively, real-time navigation ensures accurate resections and adjustments, reducing the risk of excessive bone loss or implant malpositioning.

**Figure 1:**
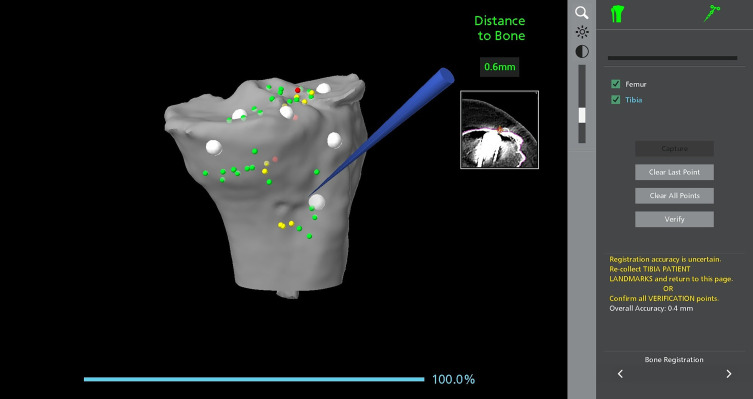
Intraoperative identification of the tibial fixation staple using the image-based robotic system. The robotic probe is utilized to locate the fixation staple, which was entirely covered by bone and not visible through direct visualization. The system provides real-time spatial feedback, displaying the distance to the bone and mapping reference points for accurate positioning. The inset image on the right shows the corresponding CT scan with the staple’s location, ensuring precise intraoperative identification.

**Figure 2:**
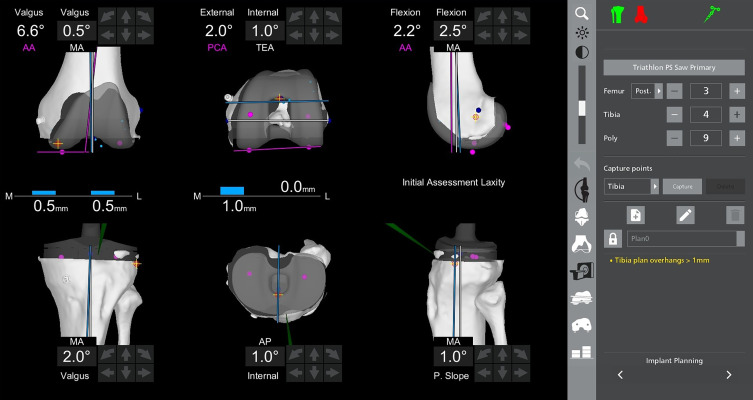
Intraoperative verification of tibial component rotation using robotic guidance. The rotation of the tibial implant is assessed with reference to Akagi’s line. The use of the probe ensures accurate rotational positioning before final implantation.

In this specific case, the tibial fixation staple was completely covered by bone, making it difficult to locate through direct visualization. Using the robotic system’s probe, it was identified position intraoperatively (Figure 1). To avoid removing the staple and creating an osseous defect, the tibial and femoral components were moved proximally by 1–2 mm, thereby preserving bone integrity while maintaining optimal alignment and stability. This strategic modification ensured that the implant positioning remained within safe biomechanical limits while avoiding unnecessary hardware removal. The rotation of the implant is verified with the probe with reference to Akagi’s line (Figure 2).

**Figure 3:**
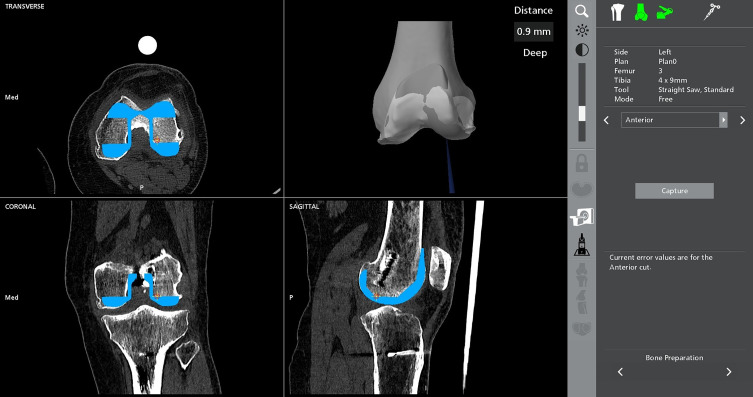
Preoperative planning of the femoral component and boxing cut using an image-based robotic system. CT-based imaging was utilized to precisely position the femoral implant while ensuring the cutting box aligned with the head of the femoral fixation screw. The robotic system provided detailed visualizations in transverse, coronal, and sagittal planes.

**Figure 4:**
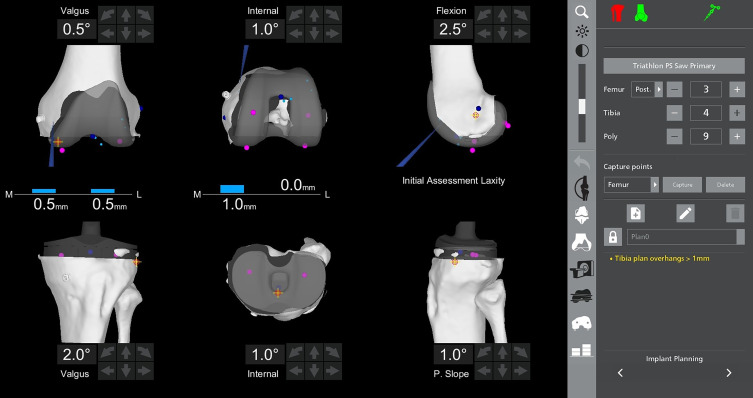
Intraoperative verification of the mediolateral positioning of the femoral implant using a robotic-assisted system. The robotic probe was utilized to confirm the precise mediolateral placement of the femoral component before proceeding with the box cut. This step ensured accurate localization of the femoral fixation screw head, preventing blind bone removal and allowing for a controlled resection while preserving bone stock.

For the femoral component, preoperative planning with the robotic system allowed to precisely position the cutting box at the head of the femoral fixation screw (Figure 3). Before proceeding with the box cut, the probe was used intraoperatively to confirm the mediolateral positioning of the femoral component, ensuring that the screw head would be accurately located during the cut. This step was crucial in preventing blind bone removal and allowed for a controlled, precise resection that avoided unnecessary bone loss (Figure 4).

### Dynamic evaluation of the anterior compartment

Dynamic evaluation of patellar tracking can be performed using the probe, allowing for real-time adjustments to optimize mediolateral tracking and anterior offset as needed [5, 10].

## Results

Table 2 presents the outcomes of the studied cohort. There was significant improvement of the KSS-knee and -function at final follow-up. No revisions or complications were recorded in this sample.

**Table 2:** The preoperative and postoperative evaluation with the Knee Society Score (KSS) is presented. Data are reported as mean values with standard deviations (SD). Independent *t*-test was used for the comparison of the mean values. Statistical significance was defined as *P* < 0.05. In italics the statistically significant values.

	Preoperative assessment	Postoperative assessment (final follow-up)	*p*-value
KSS – knee	62.88 (SD = 17.31)	93.48 (SD = 8.24)	*<0.0001*
KSS –function	78.33 (SD = 13.41)	92.2 (SD = 8.82)	*0.0001*
Knee flexion	116 (SD = 13.31)	122.08 (SD = 11.22)	0.09

## Discussion

The aim of this surgical technique was to describe the use of image-based robotic-assisted TKA in patients with a history of ACLR, integrating FA principles to optimize implant positioning and knee balance. Furthermore, a cohort of 25 patients undergoing this technique with favorable outcomes at a mean follow-up of 35.44 months was presented.

Unlike systematic alignment approaches, FA focuses on intraoperative assessment of patient-specific ligamentous laxities, allowing for individualized implant positioning that accommodates the native soft tissue envelope [8, 9, 11]. This technique provides greater adaptability in balancing flexion and extension gaps without relying on predefined coronal or sagittal alignment targets, which may lead to suboptimal outcomes in post-ACLR knees. Furthermore, the use of image-based guidance allows for precise localization of pre-existing fixation implants, enabling surgeons to either target selective extraction of interfering hardware or adjust implant positioning to avoid unnecessary removal. This flexibility helps preserve bone stock, maintain implant stability, and reduce the risk of intraoperative complications related to implant fixation interference. The possible advantages and disadvantages of the technique are presented in Table 3.

**Table 3:** Advantages and disadvantages of the use of the image-based robotic system in total knee arthroplasty in patients with previous anterior cruciate ligament reconstruction.

Advantages	Disadvantages
Precise detection of residual hardware	Radiation exposure (preoperative CT)
Targeted hardware management	Use of additional implants (pins) that could lead to system-specific complications (fractures, infections)
Individualized functional alignment (FA)	High cost
Improved soft tissue balance	Learning curve
Dynamic evaluation of the anterior compartment	Limited long-term data
Bone preservation	Closed implant system (compatible only with the manufacturer’s specific implant e.g., *Triathlon* from Stryker)
Improved implant positioning accuracy	Infrastructure and resource dependency (not available in every hospital)

FA is a relatively new technique but there are short-term studies that have demonstrated that FA in robotic-assisted TKA achieves safe coronal alignment, improved soft tissue balance, and favorable clinical outcomes [12–14]. FA has been shown to reduce the need for ligament releases while maintaining joint stability, even in complex knee morphotypes. Given that patients with a history of ACLR often experience altered knee kinematics, residual instability, and patellofemoral dysfunction, applying FA in this population could lead to more physiological post-operative outcomes compared to traditional alignment methods [1, 2]. Furthermore, FA has demonstrated comparable functional and clinical outcomes between different polyethylene insert types, highlighting its adaptability across various knee conditions [15]. FA’s soft tissue-driven approach may optimize function in post-ACLR knees by balancing stability and constraint, addressing their unique biomechanical challenges. Further studies are needed to validate these findings and assess long-term outcomes in this specific patient population.

## Conclusion

Image-based robotic-assisted TKA with FA offers a tailored approach for post-ACLR patients by addressing ligamentous laxities and hardware interference, potentially improving outcomes through optimized balance and preservation of native knee mechanics.

## Data Availability

Data is available upon reasonable request to the corresponding author

## Supplementary material

The supplementary material is available at https://www.sicot-j.org/10.1051/sicotj/2025025/olm. *Video 1*. Step-by-step surgical technique demonstrating the use of an image-based robotic system combined with a functional alignment strategy in patients with a history of anterior cruciate ligament (ACL) reconstruction.

## References

[1] Alessio-Mazzola M, Biavardi N, Solarino G, et al. (2024) Total knee arthroplasty after anterior cruciate ligament reconstruction: a narrative review. Ann Jt 9, 25. 39114421 10.21037/aoj-23-62 PMC11304087

[2] Wilson JM, Markos JR, Krych AJ, et al. (2023) Total knee arthroplasty in patients who had a prior anterior cruciate ligament reconstruction: balancing remains the issue. J Arthroplasty 38, S71–S76. 36801476 10.1016/j.arth.2023.02.037 PMC10461606

[3] Dretakis K, Koutserimpas C (2024) Pitfalls with the MAKO robotic-arm-assisted total knee arthroplasty. Medicina (Kaunas) 60, 262. 38399549 10.3390/medicina60020262 PMC10890000

[4] Diquattro E, Prill E, Salzmann M, et al. (2024) High three-dimensional accuracy of component placement and lower limb alignment using a robotic arm-assisted system and gap-balancing instrument in total knee arthroplasty. Knee Surg Sports Traumatol Arthrosc 32, 685–692. 38415872 10.1002/ksa.12088

[5] Koutserimpas C, Saffarini M, Bonnin M, et al. (2024) Optimizing the patellofemoral compartment in total knee arthroplasty: Is it time for dynamic assessment? Knee Surg Sports Traumatol Arthrosc 33, 387–392. 10.1002/ksa.12450. 39224026

[6] Lee JH, Kwon SC, Hwang JH, et al. (2024) Functional alignment maximises advantages of robotic arm-assisted total knee arthroplasty with better patient-reported outcomes compared to mechanical alignment. Knee Surg Sports Traumatol Arthrosc 32, 896–906. 38454836 10.1002/ksa.12120

[7] Choi BS, Kim SE, Yang M, et al. (2023) Functional alignment with robotic-arm assisted total knee arthroplasty demonstrated better patient-reported outcomes than mechanical alignment with manual total knee arthroplasty. Knee Surg Sports Traumatol Arthrosc 31, 1072–1080. 36378291 10.1007/s00167-022-07227-5

[8] Gregori P, Koutserimpas C, De Fazio A, et al. (2025) Functional knee positioning in patients with valgus deformity undergoing image-based robotic total knee arthroplasty: Surgical technique. SICOT J 11, 7. 39927688 10.1051/sicotj/2025001 PMC11809196

[9] Shatrov J, Battelier C, Sappey-Marinier E, et al. (2022) Functional alignment philosophy in total knee arthroplasty – Rationale and technique for the varus morphotype using a CT based robotic platform and individualized planning. SICOT J 8, 11. 35363136 10.1051/sicotj/2022010 PMC8973302

[10] Batailler C, Greiner S, Rekik H-L, et al. (2024) Intraoperative patellar tracking assessment during image-based robotic-assisted total knee arthroplasty: technical note and reliability study. SICOT J 10, 44. 39475330 10.1051/sicotj/2024037 PMC11523864

[11] Oussedik S, Abdel MP, Victor J, et al. (2020) Alignment in total knee arthroplasty. Bone Joint J 102-B, 276–279. 32114811 10.1302/0301-620X.102B3.BJJ-2019-1729

[12] Gregori P, Koutserimpas C, Giovanoulis V, et al. (2025) Functional alignment in robotic-assisted total knee arthroplasty for valgus deformity achieves safe coronal alignment and excellent short-term outcomes. Knee Surg Sports Traumatol Arthrosc. 10.1002/ksa.12585. PMC12104782 39821487

[13] Jeffrey M, Marchand P, Kouyoumdjian P, Coulomb R (2024) Short-term functional outcomes of robotic-assisted TKA are better with functional alignment compared to adjusted mechanical alignment. SICOT J 10, 2. 38240728 10.1051/sicotj/2024002 PMC10798231

[14] Koutserimpas C, Garibaldi R, Olivier F, et al. (2025) Tibial implant varus >3° does not adversely affect outcomes or revision rates in functionally aligned image-based robotic total knee arthroplasty in a minimum of 2-year follow-up. Knee Surg Sports Traumatol Arthrosc. 10.1002/ksa.12659. 40130488

[15] Koutserimpas C, Gregori P, Andriollo L, et al. (2025) Comparable outcomes between cruciate-substituting and posterior-stabilized inserts in robotic total knee arthroplasty under the functional alignment principles. Knee Surg Sports Traumatol Arthrosc. 10.1002/ksa.12654. 40079356

